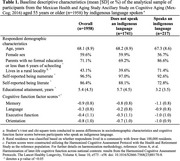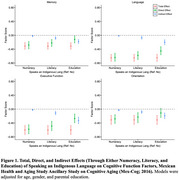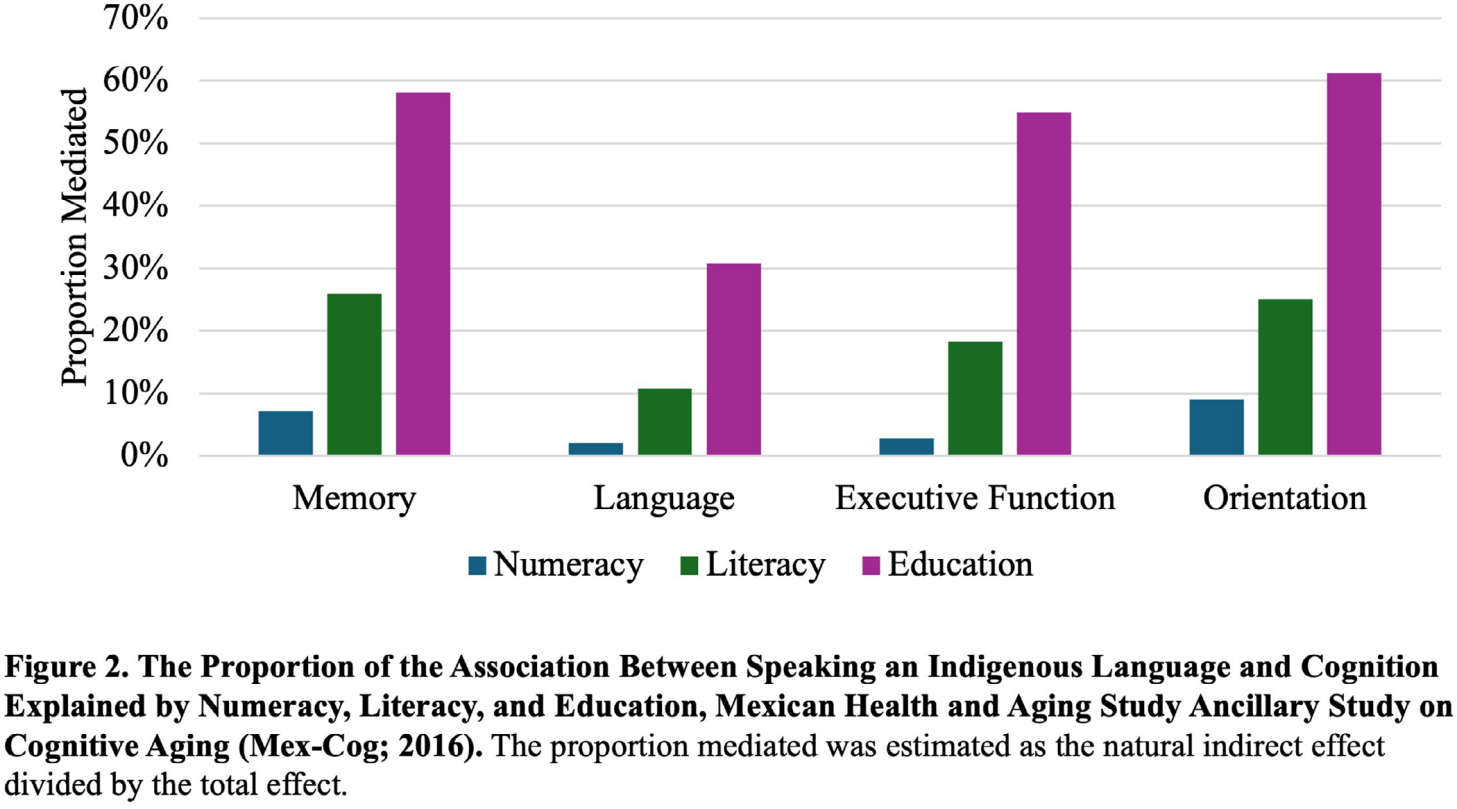# Numeracy, Literacy, and Formal Education as Mechanisms of Inequities in Late‐Life Cognitive Performance among Indigenous and Non‐Indigenous Mexican Older Adults

**DOI:** 10.1002/alz70860_106980

**Published:** 2025-12-23

**Authors:** Sirena Gutierrez, Iris Strangmann, Zachary J. Kunicki, Alexa S. Gonzalez, Gelan Ying, Jacqueline M Torres, Jennifer Weuve, Antonio Terracciano, Jet MJ Vonk, Emily M Briceno, Miguel Arce Rentería

**Affiliations:** ^1^ University of California San Francisco, San Francisco, CA, USA; ^2^ Columbia Unversity Medical Center, New York, NY, USA; ^3^ Brown University, Providence, RI, USA; ^4^ University of Houston, Houston, TX, USA; ^5^ University of Florida, Gainesville, FL, USA; ^6^ Boston University School of Public Health, Boston, MA, USA; ^7^ Florida State University, Tallahassee, FL, USA; ^8^ Memory and Aging Center, Department of Neurology, University of California San Francisco, San Francisco, CA, USA; ^9^ University of Michigan, Ann Arbor, MI, USA; ^10^ Columbia University Medical Center, New York, NY, USA

## Abstract

**Background:**

While bilingualism is generally associated with better cognitive test performance, literature suggests that Indigenous‐Spanish bilingual older adults perform worse compared to their non‐Indigenous Spanish monolingual counterparts. Older Indigenous populations in Mexico, especially in rural areas, face significant inequities in access to education and healthcare, as well as pervasive discrimination. It is unclear whether this disparity in brain aging among Indigenous adults is influenced by factors such as education, literacy, and numeracy, particularly in Mexico, where older adults generally have fewer years of schooling.

**Method:**

This study included 1958 adults ages 55+ (mean 68.1 [8.9±SD] years) from the Mexican Health and Aging Study Ancillary Study on Cognitive Aging (Mex‐Cog; 2016). Indigeneity was determined by self‐report use of an indigenous language. We employed linear regression models to estimate associations between speaking an indigenous language and domain‐specific cognitive function factor scores, adjusting for early‐life socioeconomic covariates. Utilizing mediation analysis, we decomposed the total effect of speaking an indigenous language into direct and indirect effects via numeracy, literacy, and education.

**Result:**

Among participants, 11% spoke an indigenous language. These individuals had lower educational attainment (3.2 vs. 5.7 years) and were more likely to reside in rural areas (71.4% vs. 39.6%) than those who did not speak an indigenous language. Speaking an indigenous language was associated with lower memory (β[95% CI] = ‐0.31 [‐0.40, ‐0.21]), language (‐0.65 [‐0.75, ‐0.55]), executive function (‐0.60 [‐0.73, ‐0.47]), and orientation (‐0.31 [‐0.44, ‐0.18]) scores. Under the assumptions for mediation analyses, numeracy mediated 2%‐9% (e.g., indirect effect for memory: (‐0.02 [‐0.04, 0.00])), literacy mediated 11%‐26% (e.g., indirect effect for memory (‐0.08 [‐0.12, ‐0.04])), and educational attainment mediated 31%‐61% (e.g., indirect effect for memory (‐0.18 [‐0.24, ‐0.12])) of the total effect of speaking an indigenous language on domain‐specific cognition.

**Conclusion:**

Apart from education, numeracy and literacy may also be important mechanisms underlying inequities in late‐life cognition among Indigenous populations in Mexico. Beyond educational interventions, efforts to improve numeracy and literacy may help narrow these gaps. Future research is needed to elucidate other factors, such as mid‐life health conditions and racism, that may be driving poorer cognitive outcomes among Indigenous populations.